# Enhancing Targeted Minority Class Prediction in Sentence-Level Relation Extraction

**DOI:** 10.3390/s22134911

**Published:** 2022-06-29

**Authors:** Hyeong-Ryeol Baek, Yong-Suk Choi

**Affiliations:** 1 Department of Artificial Intelligence, Hanyang University, Seoul 04763, Korea; qorgod00@hanyang.ac.kr; 2 Department of Computer Science and Engineering, Hanyang University, Seoul 04763, Korea

**Keywords:** relation extraction, minority class, data augmentation

## Abstract

Sentence-level relation extraction (RE) has a highly imbalanced data distribution that about 80% of data are labeled as negative, i.e., *no relation*; and there exist minority classes (MC) among positive labels; furthermore, some of MC instances have an incorrect label. Due to those challenges, i.e., label noise and low source availability, most of the models fail to learn MC and get zero or very low F1 scores on MCs. Previous studies, however, have rather focused on micro F1 scores and MCs have not been addressed adequately. To tackle high mis-classification errors for MCs, we introduce (1) a minority class attention module (MCAM), and (2) effective augmentation methods specialized in RE. MCAM calculates the confidence scores on MC instances to select reliable ones for augmentation, and aggregates MCs information in the process of training a model. Our experiments show that our methods achieve a state-of-the-art F1 scores on TACRED as well as enhancing minority class F1 score dramatically.

## 1. Introduction

Relation extraction (RE) is the task of identifying the semantic relation between two or more entities. For example, given the sentence “Sam[Entity1] was born in 1596[Entity2]”, the target relation-type (class) between the entities would be *person:date of birth*.

In TACRED [1] that is a widely used supervised RE dataset, we found that some classes suffer from (1) label noise that refers to the errors in labels [2] and (2) low source availability as shown in Table 1, and let denote those classes as minority classes, MCs. Due to those problems, several neural network models failed to learn MCs and got zero or very low F1 scores on MCs. For example, our experimental results showed that the average F1 test scores on MCs of C-GCN [3], KnowBERT [4], and LUKE [5] were 0%, 0%, 14.3%, respectively; the experimental results of [6] also confirmed the poor performance of 52 neural network models on MCs (details are provided in Appendix E).

Although there have been many studies that dealt with label noise or low source availability, few studies have been done to directly address MCs in RE.

As for label noise, first, manually annotated RE datasets, such as Semeval-2010-Task-8 [7], ACE 2005 (https://catalog.ldc.upenn.edu/LDC2006T06 (accessed on 25 June 2022)), and the FewRel Dataset [8], have been regarded as relatively clean, and the studies on these datasets have rarely considered the noise problem in their approach. However, a few researchers recently referred to the label noise problem in TACRED. Table 2 shows the samples of training dataset under label noise. Alt et al. [6] confirmed that the TACRED dev and test datasets were also corrupted; hence, they corrected the noisy instances and analyzed the error cases. Moreover, Stoica et al. [9] re-categorized relations in TACRED and re-annotated labels. Although those studies highlighted out the label noise problem, they focused on the dataset itself and did not deal with the learning with the noise label.

**Table 1:** Top seven classes in TACRED training dataset ordered by the level of label noise in descending order (a) and those ordered by the number of correct instances in ascending order (b). *per* and *org* are the abbreviation of person and organization, *Noise* denotes the the level of label noise for each class which is calculated by $\frac{\#\mathrm{wrong}\;\mathrm{label}}{\#\mathrm{instances}}$, and *Correct* denotes the number of correct labels for each class. Noisy labels, i.e., wrong labels are determined by the refined annotation [9]. Four classes marked in bold font suffer both of noise label and low source availability regime, i.e., MC. MC instances are totally 227 out of 68,124 training instances (0.33%) and the positive class which has most instances, 2443, is *person:title* (3.6%).

(a)	
**Class**	**Noise**
**per:country_of_death**	**83.3%**
per:countries_of_residence	80.7%
**org:shareholders**	**73.7%**
per:other_family	68.7%
**org:member_of**	**66.4%**
per:cities_of_residence	65.8%
**org:dissolved**	**65.2%**
**(b)**	
**Class**	**Correct**
**per:country_of_death**	**1**
**org:dissolved**	**8**
per:country_of_birth	15
**org:shareholders**	**20**
per:stateorprovince_of_birth	29
per:stateorprovince_of_death	33
**org:member_of**	**41**

In contrast, distant supervision for RE (DS-RE) inherently has suffered from the label noise problem and numerous studies have been conducted to solve it. Most of the existing studies mainly adopted multi-instance learning and focused on alleviating bag-level noise using sentence-level attention [10,11,12,13] or used extra information for entities [14,15]. However, no unified validation dataset for DS-RE has been proposed. Most researchers have used held-out evaluation and depended on human evaluation, which involves manually checking the subset of test instances. To tackle this problem, Gao et al. [16] published the manually annotated test set for NYT10 [17] and Wiki20 by using Wiki80 [8] that is a widely used DS-RE dataset. The study confirmed that previous models on NYT10 failed in MC prediction.

Next, as for low source availability, the imbalanced distribution is a widely acknowledged problem in RE task [18,19,20]. Negative instances, i.e., *no relation*, far exceed other instances. Moreover, even among the positive instances, the amount of clean MC instances is minimal and not sufficient for training a model. For example, the class with the most instances, i.e., *person:title* in TACRED accounts for only 3.6% of the entire training dataset and MC is much smaller, as shown in Table 1. Some studies have tackled the label sparsity in RE by adopting data augmentation [21,22,23]. However, Xu et al. [21] simply reversed the dependency path of the head and tail entities to prevent overfitting. Eyal et al. [23] validated the efficacy of their approaches on a subset of the dataset under certain scenarios. Papanikolaou et al. [22] focused on the data generation itself and required exhaustively finetuning separate models on each class. As for data augmentation, several studies have proposed masked language modeling (MLM) based data generation [24,25] for text classification. However, they do not apply to RE because they cannot guarantee the class-invariant between entities, and most labels of RE are corrupted.

In this paper, we tackle the MC problem in RE and introduced (1) a minority class attention module (**MCAM**) with the class-specific reference sentence (**Ref**), and (2) the augmentation methods particularized to RE. We applied our methods to TACRED.

The Ref is a description that narrates the definition of the keywords in the MC relation-type. Take, relation type *organization and dissolved*, for example, the Ref of it is constructed by using the definition of *origanization* and *dissolve*. We adopted only one Ref for the targeted MC, which differs from previous studies that unselectively used external knowledge for entire classes. The vector of Ref can be seen as an MC label representation. For MCAM, it is used for identifying clean instances of corresponding MC and to construct the vector that represents MCs information. In detail, MCAM calculates the reliability score by comparing the input sentence of an MC instance and its corresponding Ref, where Refs are considered as criteria for distinguishing clean instances of each MC. Based on this score, reliable samples are selected for augmentation, and additionally, the vector of MC information is constructed. Our experiments show that the proposed methods achieved a state-of-the-art (SOTA) F1 score on TACRED, as well as dramatically enhanced MC F1 scores.

In brief, the main contributions of this study are as follows:

- We propose MCAM that identifies noisy instances and improves MC prediction by constructing the vectors that represent the MCs information.
- We propose simple yet effective data generation methods particularized to RE that coordinate with MCAM and minimize the risk of relation-type change.
- Experimental results demonstrate the efficacy of the proposed approaches that enhance the overall model performance and MC prediction and is robust to spurious association.

## 2. Related Work

Distant Supervision (DS [26]) inherently has a label noise problem, and numerous approaches have been proposed to tackle it. DS involves automatic data labeling based on the assumption that if two entities in the knowledge bases (KBs) are related, the relation may hold in all sentences where these entities are found. Although DS is an effective method for generating abundant training instances by using openly available KBs (e.g., Yago, Freebase, DBpedia, Wikidata), the training instances inevitably contain significant label noise. To alleviate the label noise problem, Riedel et al. [17] and Hoffmann et al. [27] relaxed the assumption and used the multi-instance learning (MIL) [28] framework which was originally proposed to solve the task with ambiguous samples. For example, Riedel et al. [17] used the *expressed-at-least-once* assumption; it assume that at least one sentence exists where the predefined relation between the entities holds among the sentences mentioning the same entity pair. Moreover, under MIL, sentences mentioning the same entities were merged into *a bag* for each triple (relation,entity1,entity2).

Based on MIL, several researchers for DS-RE have focused on reducing the bag-level noise mainly by using an attention mechanism [10,11,12,13]. For example, Lin et al. [10] used sentence-level attention and assigned a different weight for each sentence in the same bag, and aggregated the informative representation of the sentences for the bag representation. Yuan et al. [12] used the sentence-level attention, captured the correlation among the relations, and integrated the relevant sentence bags into a super-bag to minimize bag-level noise. In addition to the attention mechanism, some studies used extra knowledge from KBs to enrich the entity and label representation to clarify the relation between entities [14,15]. For example, Ji et al. [14] used entity descriptions for the entity embedding, and Hu et al. [15] used entity descriptions for label embedding and a bag representation robust to noisy instances. However, in real-world settings, entities are infinite and the descriptions in KBs are limited; hence, they are rarely applicable. Moreover, a model depending on the entity information is prone to use the so-called shallow heuristic methods (i.e., leveraging spurious association); consequently, it is likely to fail generalization on challenging samples [29,30]. In contrast, our approaches use Refs as criteria for determining clean MC instances, which are separate from noisy instances; and adopt only one Ref for each MC relation-type that is independent of the potentially infinite entity. Moreover, this study differs from previous studies in that we selectively used external knowledge for the targeted classes only.

Regarding alleviating imbalance distribution and solving low source availability, very few studies have applied data augmentation to RE. The reason is probably the difficulty of relation-type invariance. Papanikolaou et al. [22] fine-tuned GPT-2 on each relation-type and generated augmentation dataset, which is not applicable to the RE task with many relation-types. Xu et al. [21] augmented the dataset by changing the order of the dependency path of the head and tail entities. However, the study mainly focused on preventing overfitting and not on handling imbalanced distribution. As for generating synthetic data, several studies proposed MLM based approaches [24,25]. Nevertheless, they did not consider the label noise and not guarantee the relation-type invariant. Unlike previous studies, we introduce a method for generating synthetic data particularized to RE tasks that are not exhaustive and independent of label corruption by considering the bi-directional transformer-based architecture with the target entities unchanged, i.e., preserving a relation-type.

## 3. Problem Setup

### 3.1. Task Formulation

Given a sentence $S_{i}=\left\{t_{1},t_{2},\ldots ,t_{j}\right\}$ where $t_{j}$ is the *j*-th token in the sentence $S_{i}$, the goal of RE is to predict the relation-type in a predefined label set Y between [Entity1] ($e_{1}$) and [Entity2] ($e_{2}$); our goal is to improve MC recognition. Let $M={\left\{c_{i}\right\}}_{i=1}^{n}$ denotes MC set where $c_{i}\in Y$ is one of the MCs.

### 3.2. Input Sentence Representation

As for $S_{i}$, special tokens (<s>, </s>) were added at the beginning and end of the sentence; two selected tokens (@, #) were used as entity indicators and added at the beginning and end of the entities [31,32]. Encoder of the pretrained model is used to get contextualized representation vectors as follows:

(1) $$Encoder\left(S_{i}\right)=\left[H_{t_{1}}^{S_{i}},\cdots ,H_{t_{j}}^{S_{i}}\right],$$

where $H_{t_{j}}^{S_{i}}\in R^{d}$ is the representation vector of token $t_{j}$ in the sentence $S_{i}$ and *d* is the embedding dimension of Encoder. The representation vector of sentence $S_{i}$ for the task is obtained by aggregating the representation vectors of the first token of each entity indicator:

(2) $$V_{main}^{S_{i}}=ReLU\left(W_{q}\left[H_{@}^{S_{i}};H_{\#}^{S_{i}}\right]\right),$$

where $V_{main}^{S_{i}}$ denotes the representation vector of $S_{i}$, [;] indicates concatenation and $W_{q}\in R^{d\times 2d}$. We utilize attention mechanism [33]; $V_{main}^{S_{i}}$ is used as a query vector for calculating the reliability score as shown in Equations (4) and (8).

### 3.3. Reference Sentence Representation

We used relation-type descriptions as Refs $D=\{D_{c_{1}},\cdots ,D_{c_{n}}|c_{i}\in M\}$ for each MC relation-type $c_{i}$ to set the criteria for determining clean MC instances. $c_{i}$ can have only one Ref $D_{c_{i}}$ that is composed of relation-type $c_{i}$’s keywords and their definitions. The word definitions were obtained from Wiktionary (https://www.wiktionary.org (accessed on 25 June 2022)) and Wordnet (https://wordnet.princeton.edu (accessed on 25 June 2022)), which are both open-source and publicly available.

We selected the best matching definition; however, in case a definition was too short or inadequately described the relation-type, we concatenated more than one definition with a comma (,). The entire Refs we used are provided in Appendix D.

The representation vector of $D_{c_{i}}$ is the contextualized embedding vector of special token (<s>) in $D_{c_{i}}$:

(3) $$Encoder\left(D_{c_{i}}\right)=\left[H_{t_{1}}^{D_{c_{i}}},\cdots ,H_{t_{j}}^{D_{c_{i}}}\right],$$

where $t_{1}=<s>$ and, accordingly, $H_{<s>}^{D_{c_{i}}}$ is the representation vector of $D_{c_{i}}$, i.e., label representation of $c_{i}$.

## 4. Methods

**Figure 1:**
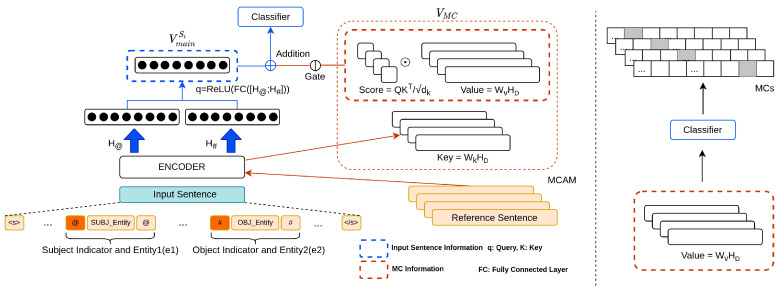
Overall architecture of our model: (**left**) aggregation of the main vector and the weighted sum of the value vectors and (**right**) incorporating MCs information into the value vector of corresponding MC. Following [31,32], special tokens (@, #) are used as entity indicators and added at before and after [Entity1] and [Entity2] tokens, respectively. We also trained a model to predict MC using its value vector alone and induced the model to align MC and its Ref vector. The representation vectors of Refs is denoted as $H_{D}=\left[H_{<s>}^{D_{c_{1}}},\cdots ,H_{<s>}^{D_{c_{n}}}\right]$.

In this section, we describe the proposed approach in detail. Figure 1 shows the overall architecture of the model. Our approaches involve three steps: (1) training the model with MCAM and attention guidance (Section 4.1), (2) filtering noisy labels and selecting the reliable instances of MC for augmentation according to the reliability score (Section 4.2), and (3) additionally training model with selective MC augmentation (Section 4.4).

### 4.1. MCAM and Classification

As shown in Figure 1, MCAM refers to operating a series of processes related to MC mainly by using the attention mechanism: (1) calculating the attention score over Refs, and (2) constructing a vector of MCs information. Here we describe how MCAM works.

#### 4.1.1. Attention Mechanism

We adopted an attention mechanism to identify noisy data and, moreover, provide a model with the vector of MCs information utilizing the concept of query, keys, and values: Query (*q*) corresponds to the representation vector of sentence $S_{i}$; and keys (K) and values (V) correspond to projections of the representation vector of Refs **D**. They can be expressed as follows:

(4) $$\begin{array}{cc} \; & \begin{array}{cc} q & =V_{main}^{S_{i}} \end{array} \end{array}$$

(5) $$\begin{array}{cc} \; & \begin{array}{cc} K & =[K_{c_{1}},\cdots ,K_{c_{n}}],\\ \; & =[W_{k}H_{<s>}^{D_{c_{1}}},\cdots ,W_{k}H_{<s>}^{D_{c_{n}}}], \end{array} \end{array}$$

(6) $$\begin{array}{cc} \; & \begin{array}{cc} V & =[V_{c_{1}},\cdots ,V_{c_{n}}],\\ \; & =[W_{v}H_{<s>}^{D_{c_{1}}},\cdots ,W_{v}H_{<s>}^{D_{c_{n}}}], \end{array} \end{array}$$

where $W_{k}\in R^{d\times d}$, $W_{v}\in R^{d\times d}$, and $K_{c_{i}}$ and $V_{c_{i}}$ is a key and value vector of $D_{c_{i}}$ respectively.

The representation vector of aggregated MCs information, $V_{MC}$, can be seen as the vector of MCs information, which is formulated as

(7) $$V_{MC}=\sum_{c_{i}\in M}\alpha_{c_{i}}\cdot V_{c_{i}},$$

where $\alpha_{c_{i}}$ is the attention score of the input sentence over $D_{c_{i}}$:

(8) $$\alpha_{c_{i}}=\langle q,K_{c_{i}}\rangle /\sqrt{d}.$$

As for $\alpha_{c_{i}}$, Softmax is not applied because it reduces the attention weights into probabilities and limits the expressibility of the vectors to which the attention weights are applied [34]. Since $\alpha_{c_{i}}$ is obtained by comparing the representation vector of an input sentence and a reference sentence, i.e., label representation, we used $|\alpha_{c_{i}}|$ as a reliability score on instances of $c_{i}$ to determine the noisy data in the process of selective augmentation (Section 4.2).

#### 4.1.2. Classification

The model output vector *O* is obtained by adding MCs information to query *q* as follows:

(9) $$O=q+g\cdot V_{MC},$$

where g∈(−1,1) denotes gate unit that regulates the flow of MC information:

(10) $$g=\tanh(W_{g}\cdot q),$$

where $W_{g}\in R^{1\times d}$.

Given $S_{i}$ and D, to compute the probability on each relation-type, the projection of the output vector is fed into a softmax layer as shown below:

(11) $$P\left(r|S_{i},D;\theta \right)={\mathrm{Softmax}}_{r}\left(W_{o}O\right),$$

where P(r|·;θ) is the prediction probability on relation-type r∈Y of a model which is parameterized by θ, $W_{o}\in R^{L\times d}$ and *L* is the total number of relation-types. Accordingly, given N samples, cross entropy loss function $L_{clf}$ can be formulated as:

(12) $$L_{clf}=-\sum_{i=1}^{N}\log P(y_{i}|S_{i},D;\theta ),$$

where $y_{i}$ is an annotated label on $S_{i}$.

#### 4.1.3. Attention Guidance

Attention guidance is to make a model that connects the Ref and its corresponding MC. Without explicit guidance, it is hard for a model to match the plain text, Ref, to the corresponding MC. To solve this problem, we trained the classifier to predict each MC using the corresponding Ref alone (i.e., without input sentence) through the following loss function $L_{ref}$, which enables us to directly incorporate MC $c_{i}$ label information into $V_{c_{i}}$ as follows:

(13) $$\begin{array}{cc} \; & \begin{array}{cc} L_{ref} & =-\sum_{c_{i}\in M}\log P(c_{i}|D_{c_{i}};\theta ),\; \end{array} \end{array}$$

(14) $$\begin{array}{cc} \; & \begin{array}{cc} P(c_{i}|D_{c_{i}};\theta ) & ={\mathrm{Softmax}}_{c_{i}}\left(W_{o}V_{c_{i}}\right).\; \end{array} \end{array}$$

As shown in Equation (14), it differs from Equation (11) in that Equation (14) does not use $S_{i}$ and the entire Refs D, but instead uses only one Ref, $D_{c_{i}}$. An illustrative example is provided in Appendix C.

#### 4.1.4. *Self* Attention Guidance

In addition to attention guidance, we utilized *self* attention guidance to obtain more accurate attention scores which are used to determine the noisy data.

It is inspired by the study of [35] that uses this method to minimize the prediction score of the ground truth class after a pixel-level segmentation mask is applied to the specific area that obtains a higher attention score than a predefined threshold. This approach encourages the model to learn that the masked area is important for predicting the corresponding class and extracting more complete attention maps. We modified this method and adapted it to our model when the instance belongs to M.

The processes are as follows: (1) given y=k (k∈M), flipping the sign of attention weight on V in Equation (6) and calculating the output vector:

(15) $$O^{\prime }=q+g\cdot \sum_{c_{i}\in M}\left(-\alpha_{c_{i}}\right)\cdot V_{c_{i}},$$

and (2) minimize the corresponding prediction score which is denoted as $L_{flip}$ as given below:

(16) $$L_{flip}={\mathrm{Softmax}}_{k}\left(W_{o}O^{\prime }\right).$$

Therefore, our objective function is $L=L_{clf}+L_{ref}+L_{flip}$.

### 4.2. Selective Data Augmentation

**Figure 2:**
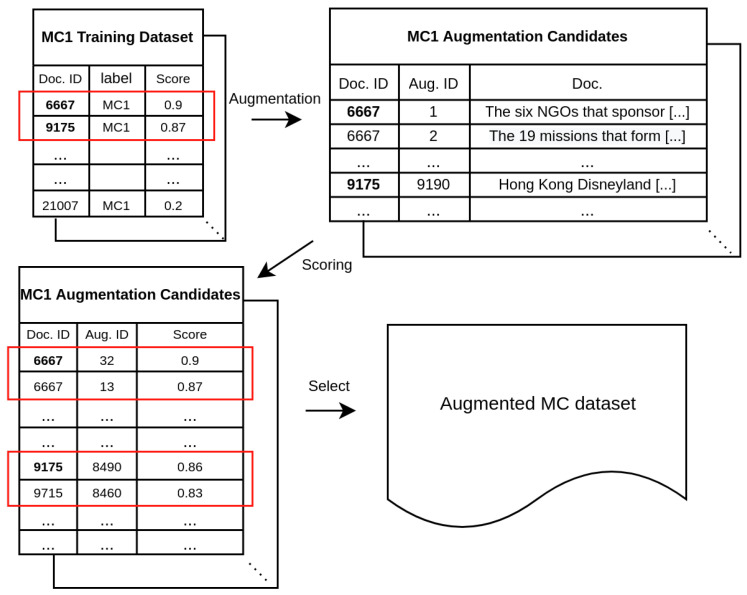
Workflow for the selective augmentation of MC.

As illustrated in Figure 2, we selected the reliable instances of MCs according to the following procedure: (1) arranging the MC instances in descending order according to the reliability score on the corresponding Ref, (2) selecting the higher *m*% instances, i.e., reliable instances, (3) generating synthetic data and re-calculating reliability scores on them, and (4) taking a subset of the synthetic data into a training dataset based on those scores.

In step (4), the size of the augmentation is a hyper-parameter and illustrative experiments are provided in Section 6.2. In step (2), regarding *m*% we determined it by estimating the level of valid annotation on relation-type $c_{k}$. Let denote it as $\rho_{c_{k}}$ and, then, $1-\rho_{c_{k}}$ represents the level of label noise. $\rho_{c_{k}}$ is derived by calculating the number of instances aligning with the corresponding Ref $D_{c_{k}}$:

(17) $$\rho_{c_{k}}=\frac{\sum_{i\in N(c_{k})}𝟙[{\mathrm{argmax}}_{c_{j}\in M}|\alpha_{c_{j}}\left(S_{i}\right)\left|\right]}{\left|N(c_{k})\right|},$$

where $N(c_{k})$ is the index set of $c_{k}$ instances, $|\alpha_{c_{j}}\left(S_{i}\right)|$ is the absolute value of attention score of sentence $S_{i}$ over $D_{c_{j}}$, and 𝟙[·] is the indicator function that is equal to 1 when given $y_{i}=c_{k}$ the value inside the function is $c_{k}$ or 0 otherwise. We averaged $\rho_{c_{k}}$ of each MC (i.e., $\frac{1}{\left|M\right|}\sum_{c_{k}\in M}\rho_{c_{k}}$) to determine the size of reliable instance per MC.

### 4.3. Generating Synthetic Data

Regarding the step (3) in Section 4.2, we designed a method for generating synthetic data particularized to RE that preserves the relation-type between entities, i.e., label-invariant augmentation. We utilized MLM and conducted following the steps: (1) finetuning pretrained model on a training dataset with MLM task, (2) after completing finetuning, incrementally masking a token with the special token, [MASK], from the beginning to the end of the target sentence except for entity tokens, (3) inferencing the masked token with the finetuned model, (4) replacing it by using *top-k random sampling* strategy [36], and (5) repeatedly implementing step (2) to (4) and generating K′ synthetic data per reliable instance (we set $K^{\prime }$ as 300).

This approach can introduce data diversity, minimize the risk of relation-type change and is independent of label noise, because the model learns the token distribution around the target entities in the process of finetuning that is irrelevant to relation-type and bidirectional-attention models, such as BERT, can exploit preserved target entities to predict the masked token. The pseudo-code for generating synthetic data is provided in Algorithm 1.
**Algorithm 1** Pseudo Code for Generating Augmentation Candidates**Data**: The dataset $T_{clean}$ consisting of selected and reliable MC instances**Parameter**: Learned masked language model parameters $\hat{\theta }$**Initialize**: An augmentation set $T_{aug}\leftarrow \left\{\right\}$
  
    **for** 
$S_{i}\in T_{clean}$ **do**
        count←0        **while** count≤K′ **do**            $S_{i}^{\prime }\leftarrow Copy\left(S_{i}\right)$            **for** $t_{j}\in S_{i}$ **do**                **if** j∉EntitySpan **then**                    $S_{i}^{\prime }\leftarrow Replace\left(S_{i}^{\prime },t_{j},\left[\mathrm{MASK}\right]\right)$                    $\hat{tj}\leftarrow TopKSampling\left(argmax_{\hat{tj}}Pr\left(\hat{t_{ji}};\hat{\theta }\right)\right)$                    $S_{i}^{\prime }\leftarrow Replace\left(S_{i}^{\prime },\left[\mathrm{MASK}\right],\hat{tj}\right)$                **else**                    Continue                **end if**           **end for**           $T_{aug}\leftarrow T_{aug}\cup S_{i}^{\prime }$            count←count+1        **end while**    **end for**


### 4.4. Additional Training with MC Augmentation

To improve the model performance on predicting MCs, we trained the model with more epochs with the augmented dataset and adapted two additional training strategies [37,38]: (1) freezing the backbone model parameters to preserve the information learned from the main training process, and (2) selectively training the instances on which the model’s prediction probability is lower than the predefined threshold to prevent overfitting (details are provided in Appendix A). Additionally, label smoothing regularization [39] (LSR) was applied throughout the additional training process to mitigate the effect of label noise and for the calibration [40,41] of which the parameter ϵ was set as the averaged the level of label noise calculated from Equation (17). Thus the objective function for the additional training is $L^{\prime }=LSR\left(L_{clf};\epsilon \right)+LSR\left(L_{ref};\epsilon \right)+L_{flip}$ where LSR(·;ϵ) is LSR operation parameterized by ϵ.

## 5. Experiments

In the following sections, we evaluate the proposed methods. Our code is publicly available at https://github.com/henry-paik/EnhancingREMC (accessed on 25 June 2022).

### 5.1. Dataset and Baselines

We trained our models on the training dataset of **TACRED** [1] for which statistics is provided in Table 3. Experiments were performed on the test dataset of TACRED and two extended TACRED datasets [6,29]. Alt et al. [6] corrected wrong labels and published a revised version of TACRED dev and test datasets. This dataset is denoted as revised TACRED (**Rev-TACRED**). Rosenman et al. [29] consists of challenging and adversarial samples designed to verify the robustness of models to the so-called *shallow heuristic methods*, e.g., highly dependent on the existence of specific words or entity types in the sentence while not understanding the actual relation between entities. This is denoted as challenging RE (**CRE**).

We compared our model with the following models: (1) C-GCN [3], (2) LUKE [5], (3) SpanBERT [42], (4) KnowBERT [4], (5) RoBERTa-large [43], and (6) RE-marker [32].

### 5.2. Metrics

In addition to using a micro F1 score (**F1**), we used a macro F1 score (**Ma. F1**) that is the average of the per-class F1 scores. Unlike **F1**, **Ma. F1** is insensitive to the majority classes. For Rev-TACRED, we additionally adopted **MC F1** and a weighted MC F1 score (**W. MC F1**). **MC F1** is calculated on four MCs while other relation-types are neglected to calculate the model performance on MCs alone. **W. MC F1** is an instance-wise weighted micro F1 score on the MC instances to measure the model performance on difficult samples among MCs, where the weight, from 0 to 1, is assigned to each instance according to the difficulty calculated by the seed models from [6]. Details are provided in Table A4.

We also adopted positive accuracy (**Acc+**) and negative accuracy (**Acc−**) on CRE that [29] developed for measuring the robustness against leveraging spurious association. Let’s take the following two sentences, for example:

- S1: **Ed[e1]** was born in **1561[e2]**, the son of John, a carpenter, and his wife Mary.
- S2: Ed was born in **1561[e2]**, the son of **John[e1]**, a carpenter, and his wife Mary.

If a model depends on leveraging spurious association, even though it can correctly classify S1 as *person:date of birth*, it is very likely to predict that the relation still holds in S2, which is incorrect. Acc- is calculated on the adversarial instance (S2) where the relation does not hold anymore. Thus, a high Acc- value suggests that a model is robust to the so-called heuristic methods, understanding the actual relation between entities.

### 5.3. Implementation Details

In this experiment, we built our model, RE-MC, by equipping RoBERTa-large with MCAM; trained it with nine settings of data augmentation varying scale factor *N* and minimum proportion *S* of the token replacements to the entire tokens. We set N={2,4,8} by which the original size of MC (227) was multiplied, i.e., total augmentation size would be 454, 908, and 1816, respectively, which are evenly distributed to each MC; *S* was set as S={0.1,0.2,0.3}, which is a constraint on MLM with the pretrained model that should be satisfied. Empirical analysis of *N* and *S* is provided in Section 6.2.

We trained RE-MC on three different random seeds, and selected one of them that yielded the median F1 on Rev-TACRED dev. In the following sections, we report the results of the model trained on that seed. As for generating synthetic dataset, we finetuned RoBERTa-base on the TACRED training dataset for 100 epochs. Other settings are provided in Appendix B.

As described in Table 1, the targeted MCs for our methods to improve are as follows: *per:country of death* (c1), *org:member of* (c_2_), *org:dissolved* (c_3_), and *org:shareholders* (c_4_).

### 5.4. Results

Table 4 presents the test results on TACRED and Rev-TACRED. The results show the SOTA performance on the overall metrics, not only for MC, which is meaningful results in that our methods are robust to be biased either toward MCs nor majority classes. Compared with RE-marker our model is based on, we can see that MCAM and selective augmentation improved the overall model performance (F1 75.4% and 84.8% on TACRED and Rev-TACRED respectively), which indicates that our approaches can be applied to other base models to reinforce MC prediction, i.e., model-agnotic in that we simply added MCAM and selective augmentation to RE-marker to build our model. Subsequently, regarding W. MC F1 RE-MC outperforms the other models by a large margin of at least Δ26.9%, demonstrating the efficacy of our approaches to dealing with MC. RE-MC _(*N* = 8, *S* = 0.1)_, especially, can be the most effective settings for dealing with MC (49.1% and 71.4% on MC F1 and W. MC F1), even though it might be a relatively limited increase in the overall F1 compared to other settings.

Furthermore, as shown in Table 5, the proposed approach is robust to heuristic methods, i.e., rarely leveraging spurious association, indicating that our augmentation strategy is good for token perturbation and relation-type invariants.

### 5.5. Significance Test

For MC scores, we conducted a significance test because the number of MC instances in TACRED-Rev test set was small, 18 ($c_{1}$: 10, $c_{2}$: 4, $c_{3}$: 1, $c_{4}$: 3). To increase the quantity of MC instances, we additionally took the refined annotation from [9] after manually inspecting the annotations. Finally, the significance test was conducted using total 33 MC instances ($c_{1}$: 14, $c_{2}$: 4, $c_{3}$: 4, $c_{4}$: 11). We did bootstrapping 100,000 times, for each size of 33, and calculated MC F1.

**Figure 3:**
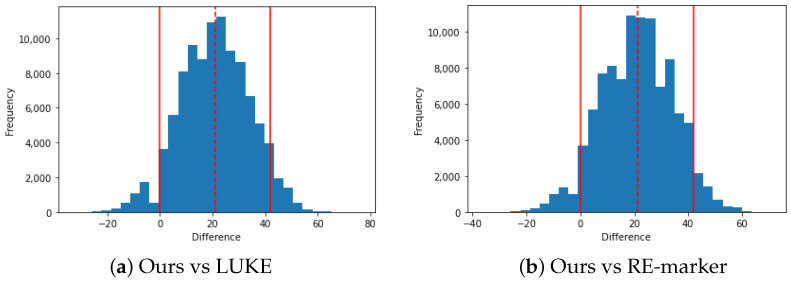
Distribution of the bootstrapping results. We calculated the difference between MC F1 scores of ours and LUKE (**a**) and RE-marker (**b**), respectively. *X*-axis represents the difference between MC F1 scores and *Y*-axis represents the frequency. The value of lower bound and upper bound (solid line), and median (dotted line) under 90% confidence level is marked in the figures.

The results of significance test between RE-MC _(*N* = 2, *S* = 0.1)_ (bootstrapping mean is 42.3) and two main competitive models, i.e., LUKE and RE-Marker (bootstrapping means are both 21.1), show that the difference is significant at 90% confidence level as shown in Table 6 and Figure 3. Table 6 shows the lower and upper bound of 90% confidence interval and Figure 3 shows the distribution of bootstrapping results of the difference between MC F1 scores of ours and RE-marker and LUKE, respectively.

## 6. Analysis

### 6.1. Ablation Study

Table 7 shows the efficacy of our methods, such as selective augmentation, additional training, and LSR; removal of each component causes the significant performance deterioration on MC prediction. As for selective augmentation, it leads to significant improvements in MC prediction (MC F1 9.1 → 47.1), which indicates that it is the critical component for MC prediction. The removal of additional training shows the deterioration of the MC prediction performance (MC F1 9.1 → 0). We can also see that LSR contributes to improving MC prediction (MC F1 27.6 → 47.1).

### 6.2. Augmentation Size and Token Replacements

**Figure 4:**
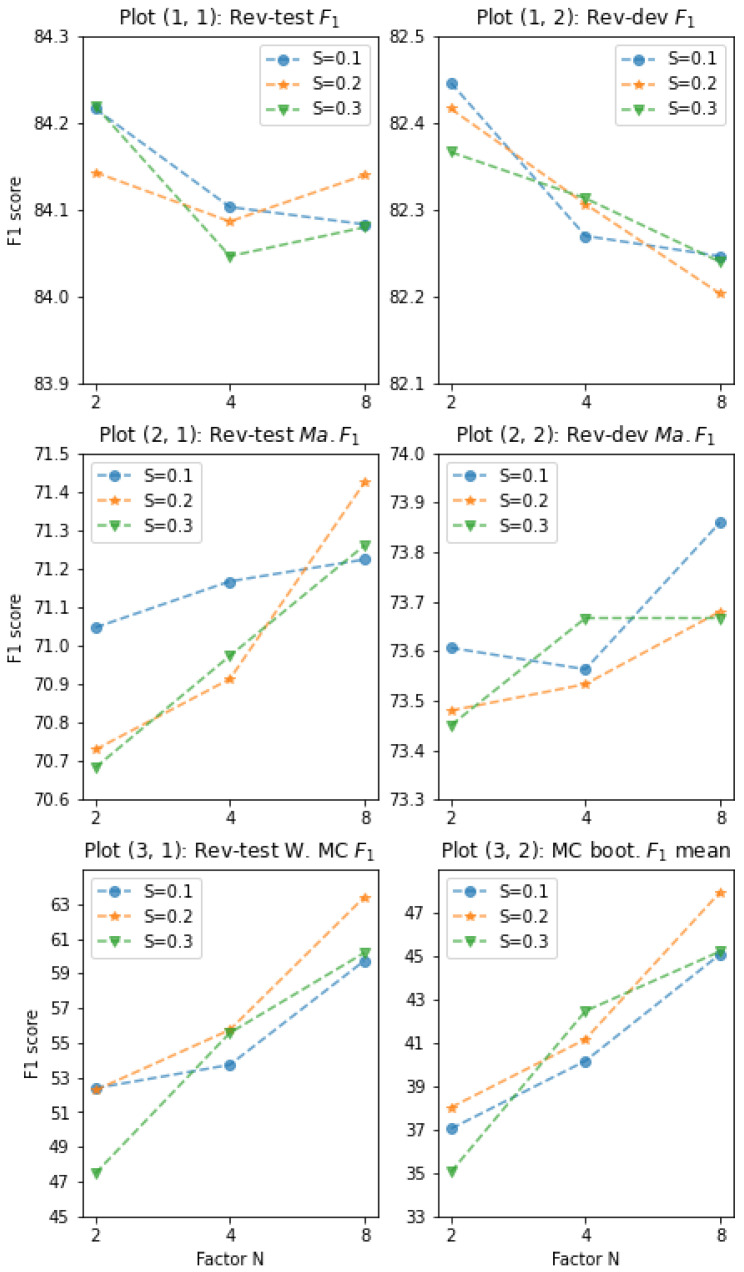
Augmentation settings and F1 scores on Rev-TACRED test and dev datasets. *Y*-axis is F1; *X*-axis is scale factor *N*; legend *S* is the proportion of the token replacements; and MC boot. F1 in plot (3, 2) denotes the bootstrap mean of MC F1 score.

To analyze the effects of the augmentation size and token replacements, we set nine different MC augmentation datasets by varying the scale factor *N* = {2,4,8} and the minimum proportion of token replacements *S* = {0.1,0.2,0.3} where the actual average proportion was 0.21, 0.28, and 0.35, respectively. Figure 4 shows the results of the average scores of 30 models for each setting, which were the top ten models from three different random seeds, respectively, based on Rev-TACRED dev F1. Following the experimental results in Figure 4, we reported the scores of the optimal parameter-combination in Table 4 (i.e., *N* = 2, *S* = 0.1; *N* = 4, *S* = 0.3; and *N* = 8, *S* = 0.1).

As shown in plot (1, 1), the entire augmentation settings are effective, and the values are consistently higher than those of other base models shown in Table 4 (minimum F1 in plot (1, 1) is greater than 84%). For MCs, in plot (3, 1) and (3, 2), we can clearly see that MC prediction performance increases dramatically as *N* becomes larger, especially when *S* = 0.3. For example, given *S* = 0.3, the maximum differences are yielded between the case of *N* = 2 and *N* = 8 in plot (3, 1), Δ13%, and (3, 2), Δ10.2%. It indicates that a low MC F1 is attributed to the low source availability, and our augmentation approach functions properly.

Regarding F1 and Ma. F1 in plots (1, 1) and (2, 1), the trends are contrary to each other: the former decreases and the latter increases as *N* becomes larger. However, owing to greater improvements in MC as shown in plot (3, 1), the drops on F1 are offset by the rapid increase in Ma. F1, which is evident when comparing the slopes in plots (1, 1) and (2, 1).

## 7. Conclusions

This study demonstrated that MC prediction in TACRED under label noise and low source regimes could be improved by using MCAM with Refs and selective augmentation. The experimental results showed that the proposed methods significantly improved the overall performance and MC prediction. Moreover, these methods are also robust to heuristic methods. While our approaches proved efficacy in dealing with MC for RE, we should further extend the usage of MCAM architecture to other tasks where MC problems prevail but text Ref is not available. Our future work includes finding an appropriate proxy of Ref and strategies to embed MCs information for other tasks.

## Figures and Tables

**Table 2:** Examples of traininig dataset from TACRED. The relation between [Entity1] and [Entity2] is annotated as shown in the TACRED label column.

Sentence	TACRED Label	Correct?
Kaiser’s parents had emigrated in 1905 from Ukraine, then part of **Russia[Entity2]**, where **his[Entity1]** four oldest siblings were born.	per:country of death	No
The president told **ABC radio[Entity1]**’s Sunday Profile program that violence in his country since its independence **five years ago[Entity2]** has been because the nation has had to begin from scratch...	org:dissolved	No
**It[Entity1]** was disbanded in **2003[Entity2]**.	org:dissolved	Yes

**Table 3:** Training dataset statistics. We list the number of relations (# Rel), MC instances (# MC), and *no relation* instances (# N/A) with the percentage.

Datasets	# Rel	# MC (%)	# N/A (%)	# Total
TACRED	42	227 (0.33)	55,112 (81)	68,124

**Table 4:** The test scores on TACRED and Rev-TACRED. Results with * are from [6].

Data	Model	F1	Ma. F1	MC F1	W. MC F1
TACRED	C-GCN	67.3	49.5	17.4	-
	SpanBERT *	70.8	56.1	19.2	-
	KnowBERT *	71.5	57.6	12.5	-
	LUKE	72.7	58.9	3.8	-
	RE-marker	74.5	62	12.2	-
	RE-MC _(*N* = 2, *S* = 0.1)_	75.1	62.1	24.1	-
	RE-MC _(*N* = 4, *S* = 0.3)_	**75.4**	**63.4**	**27.6**	-
	RE-MC _(*N* = 8, *S* = 0.1)_	74.6	62.5	26.9	-
Rev-TACRED	C-GCN	74.8	55.5	0	0
	SpanBERT *	78	63.7	21.4	16.6
	KnowBERT *	79.3	63.4	0	0
	LUKE	81.5	67	14.3	11
	RE-marker	82.9	70.8	24	24.9
	RE-MC _(*N* = 2, *S* = 0.1)_	**84.8**	71.8	47.1	53.3
	RE-MC _(*N* = 4, *S* = 0.3)_	84.7	**72**	44	51.8
	RE-MC _(*N* = 8, *S* = 0.1)_	83.3	70	**49.1**	**71.4**

**Table 5:** The test scores on CRE. A model with a higher Acc− score, and a smaller gap (Diff.) between Acc+ and Acc− is considered more robust to heuristic methods, i.e., spurious association. Results with ${}^{†}$ are from [29].

Model	Acc	Acc+	Acc−	Diff.
SpanBERT ${}^{†}$	63.5	**89.7**	42.5	47.2
KnowBERT ${}^{†}$	72.4	84.2	62.9	21.3
LUKE	**80.8**	87.3	75.5	11.8
RE-marker	78.6	87.5	71.4	16.1
RE-MC_(*N* = 2, *S* = 0.1)_	80.2	84.8	**76.6**	**8.2**

**Table 6:** 90% confidence interval of the differences between MC F1 scores of models. L.B., U.B. and M denotes the lower bound, upper bound and median value, respectively.

	L.B.	U.B.	M
Ours—LUKE	0	42.9	21.2
Ours—RE-Marker	0	41.9	21.2

**Table 7:** Performance comparison for ablation study. *w/o Aug* denotes the removal of augmentation; *w/o Add* denotes the removal of additional training; and *w/o LSR* denotes removal of LSR when additional training.

Model	F1	Ma. F1	MC F1
RE-MC _(*N* = 2, *S* = 0.1)_	**84.8**	**71.8**	**47.1**
*w/o Aug*	84.6	70.9	9.1
*w/o Aug w/o Add*	83.3	68	0
*w/o LSR*	84.2	70	27.6

**Figure A1:**
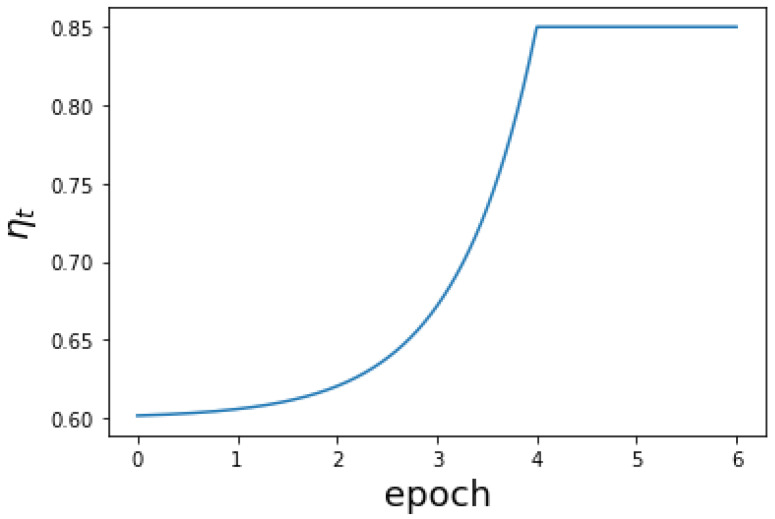
Exponential schedule. We introduce maximum $\eta_{max}$ and minimum $\eta_{min}$ threshold, run maximum 6 epochs and set the epoch *E* as 4 where schedule reach $\eta_{max}$. Empirically, exponential schedule is suitable for the model, in particular, which is suffering from learning MCs pattern. We set $\eta_{t}=\min\left(\left(1-exp\left(-\frac{t}{T}\times 5\right)\right)\times \left(\eta_{max}-\eta_{min}\right)+\eta_{min},\eta_{max}\right)$, where *T* is the product of the total steps per epoch and *E*.

**Figure A2:**
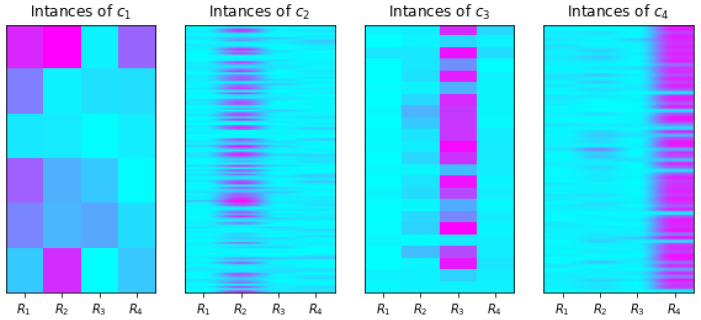
Heatmaps of the absolute attention scores for each MC instance in TACRED training dataset over value vectors of reference sentences. The *Y*-axis represents an instance, the *X*-axis represents a reference sentence, and the value is the reliability score. The expected heat map of the ideal dataset and MCAM is showing a high value (almost purple) on the *i*-th column of the *i*-th figure where the scores of the $c_{i}$ instances are plotted.

## Appendix A. Additional Training

Xie et al. [38] introduced Training Signal Annealing (TSA) and gradually increased the schedule of confidence threshold $\eta_{t}$ at every step *t*. We modified the schedule and adapted it to our additional trainining as shown in Figure A1.

## Appendix B. Experimental Settings

Training and experiments are conducted on a Ubuntu20.04 server with Intel (R) Core (TM) i9-10980XE CPU and GeForce RTX 3090 GPU. For TACRED, we used RoBERTa-large [43] as a backbone model, used learning rate 5 × 10 ${}^{-6}$ and batch size of 4 for initial training, and batch size of 4 and learning rate 5 × 10 ${}^{-6}$ for additional training. The checkpoint of backbone model was obtained from https://huggingface.co/roberta-large (accessed on 25 June 2022), the number of parameters is 355M, and that of ours is 357M.

The hyper parameter settings for RE-MC _(*N* = 2, *S* = 0.1)_ are as shown in Table A1 where the parameter of label smoothing was determined by using Equation (17) but statistical approaches to ratio estimation [44] or noise estimation [45] also can be used. The number of augmentation dataset are provided in Table A2 and MCs distribution is provided in Table A3.

We searched hyperparameters as follows:

- learning rate: 1 × 10 ${}^{-5}$, 5 × 10 ${}^{-6}$, 1 × 10 ${}^{-6}$;
- batch size: 2, 4, 6.

**Table A1:** Hyper parameters. CE denotes $L_{clf}$ and AG denotes $L_{ref}$.

Name	Value
Maximum word length	512
Mini batch size	4
Learning rate	5 × 10 ${}^{-6}$
Optimizer	AdamW
Warmup steps	the first 10% of steps of the first epoch
Weight decay	1 × 10 ${}^{-4}$
Initial training epochs	5
Additional training epochs	6
Label smoothing $\epsilon_{1}$ (CE)	0.3
Label smoothing $\epsilon_{2}$ (AG)	0.3

**Table A2:** Selected model implementation details for TACRED.

	(*N* = 2, *S* = 0.1)	(*N* = 4, *S* = 0.3)	(*N* = 8, *S* = 0.1)
# Aug.	429	901	1814
$c_{1}$	114	228	454
$c_{2}$	106	212	462
$c_{3}$	99	231	442
$c_{4}$	110	230	456
# Total	68,424	68,896	69,789

**Table A3:** MCs distribution. Train and Test is that of TACRED and R- indicates Revised TACRED. Aug. indicates augmentation which of values was added to the original training dataset for our final model (RE-MC).

	Train	Test	R-Test	R-Dev
per:country of death	6	9	10	47
org:member of	122	18	4	7
org:dissolved	23	2	1	1
org:shareholders	76	13	3	35

## Appendix C. Attention Guidance

Figure A2 shows that the model assign a high reliability score to the intended reference vector of each MC. The instance with a blue-colored cell on the corresponding value vector of reference sentence is likely to be an error in the label with higher probability, i.e., an incorrect annotation.

## Appendix D. Reference Sentence

We provide reference sentences for TACRED as follows:

- **org:member_of**: ‘the relation is “organization and member of”. organization: a group of people or other legal entities with an explicit purpose and written rules. member: one who officially belongs to a group, a part of a whole, one of the persons who compose a social group (especially individuals who have joined and participate in a group organization). of: having a partitive effect, introduces the whole for which is indicated only the specified part or segment, from among, indicates a given part.’
- **org:dissolved**: ‘the relation is “organization and dissolved”. organization: a group of people or other legal entities with an explicit purpose and written rules. dissolve: stop functioning or cohering as a unit, to terminate a union of multiple members actively, as by disbanding, to destroy, make disappear.’
- **per:country_of_death**: ‘the relation is “person and country of death”. person: an individual, usually a human being. country: the territory of a nation, especially an independent nation state or formerly independent nation, a political entity asserting ultimate authority over a geographical area, a sovereign state, a politically organized body of people under a single government. death: the cessation of life and all associated processes, the end of an organism’s existence as an entity independent from its environment and its return to an inert, nonliving state, the event of dying or departure from life’.
- **org:shareholders**: ‘the relation is “organization and shareholders”. organization: a group of people or other legal entities with an explicit purpose and written rules. shareholder: one who owns shares of stock in a corporation, someone who holds shares of stock in a corporation.’

## Appendix E. Model Performance on MCs

Alt et al. [6] tested 52 RE models on Rev-TACRED test; we used the experimental results to calculate the average number of models that correctly predict for each class $\left(\frac{\#\mathrm{Correct}\;\mathrm{Prediction}}{\#\mathrm{RE}\;\mathrm{Models}}\right)$. For example, regarding org:dissolved, on average 0.5 RE models correctly do classification on the instances belong to org:dissolved. As shown in Table A4, models generally failed predict MCs.

As for the metric W. MC F1, instance-wise weight of difficulty is calculated by the same experimental source that Table A4 is based on. For example, the instance below belongs to *per:country_of_death* but none of 52 models correctly predicted, and hence the weight of this instance is one (i.e., 52/52):

- *They say [...] **[Entity1]** died late Saturday [...] in southern **Finland [Entity2]**, while [...]*
  where we omitted the name and unimportant tokens.

**Table A4:** The average number of models that correctly predict for each class.

Relation Type	Average Number of Models
per:country_of_death	0
org:member_of	0.1
org:dissolved	0.5
org:shareholders	1.3
per:country_of_birth	1.4
org:members	1.7
per:alternate_names	2.5
per:other_family	4.7
org:parents	10.6
per:stateorprovince_of_death	12.1
org:subsidiaries	13.5
per:city_of_death	14.9
per:cause_of_death	16.3
org:founded_by	17.8
per:date_of_death	18.3
per:city_of_birth	19.6
org:country_of_headquarters	20.3
per:children	20.4
org:political/religious_affiliation	21
per:parents	21.2
per:countries_of_residence	22.2
per:religion	23.6
per:siblings	23.9
org:number_of_employees/members	25.1
per:stateorprovinces_of_residence	25.2
per:stateorprovince_of_birth	25.3
per:cities_of_residence	25.3
per:schools_attended	27.4
per:origin	29.2
per:spouse	30
per:employee_of	30.9
org:stateorprovince_of_headquarters	34.7
org:city_of_headquarters	35.2
per:date_of_birth	38.1
per:charges	38.4
org:website	41.5
org:alternate_names	41.7
org:founded	42.1
org:top_members/employees	42.4
per:title	42.5
per:age	44.9
no_relation	48.4
